# Supplementary material for: Unmodulated 40 Hz Stimulation as a Therapeutic Strategy for Aging: Improvements in Metabolism, Frailty, and Cognitive Function in Senescence-Accelerated Prone 10 Mice
Source: Biomolecules. 2024 Aug 28;14(9):1079. doi: 10.3390/biom14091079 (PMC11429768; doi:10.3390/biom14091079)
Supplement: Supplementary file 1 [file biomolecules-14-01079-s001.zip › biomolecules-3125015-supplementary.pdf]

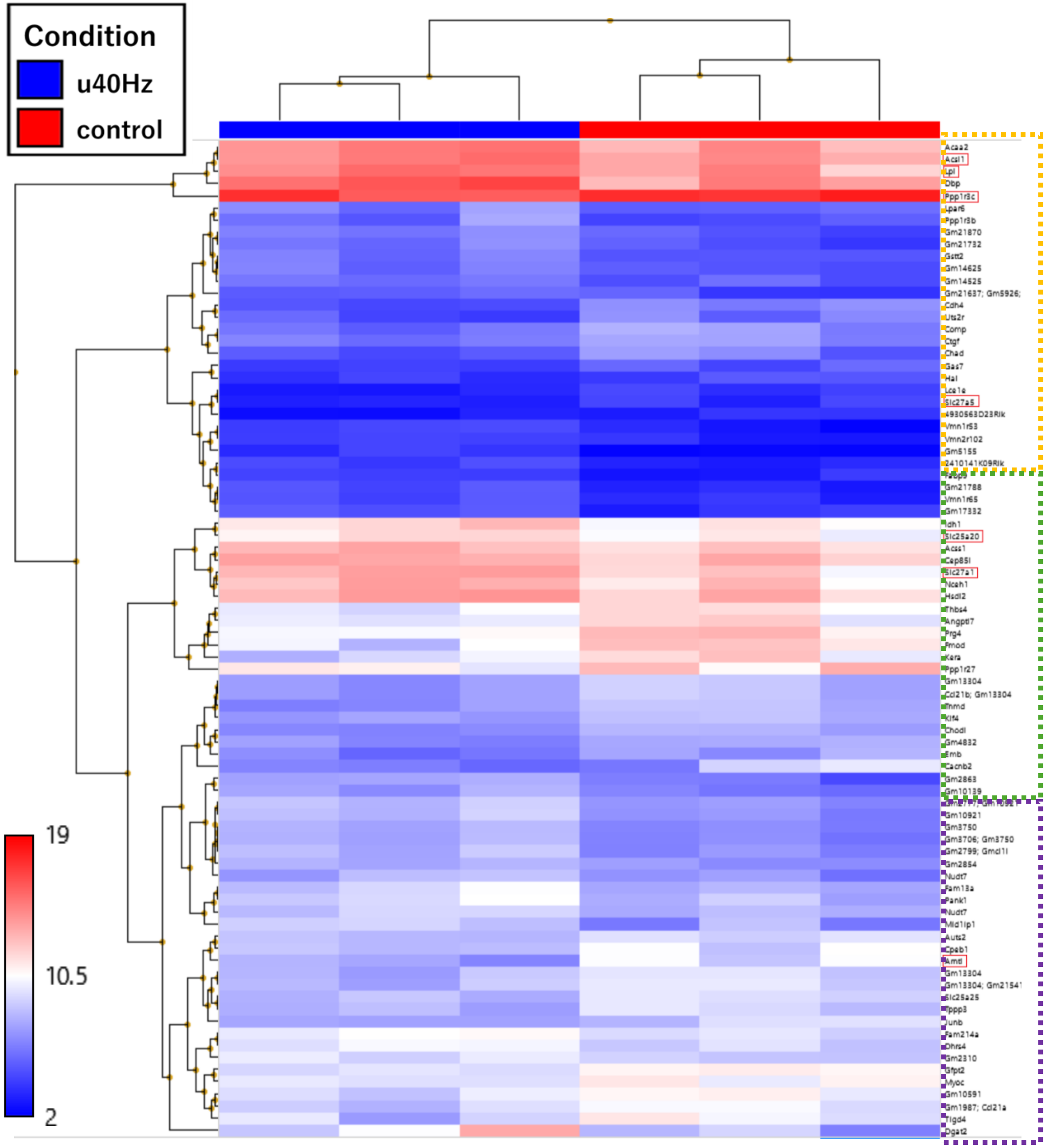

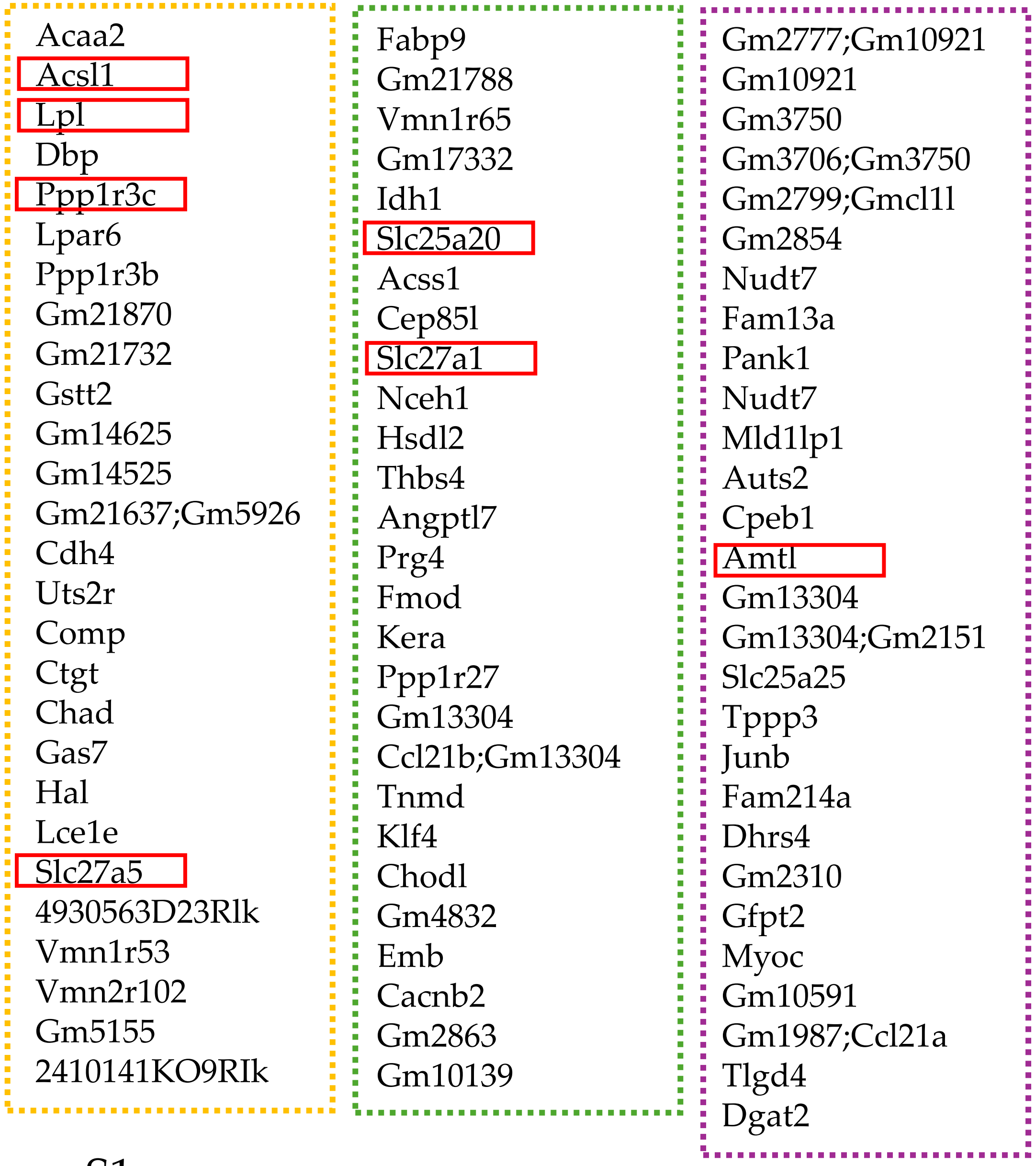

Figure.S1:
Heatmap of cluster analysis from microarray data of muscles in 40 Hz stimulation and control groups

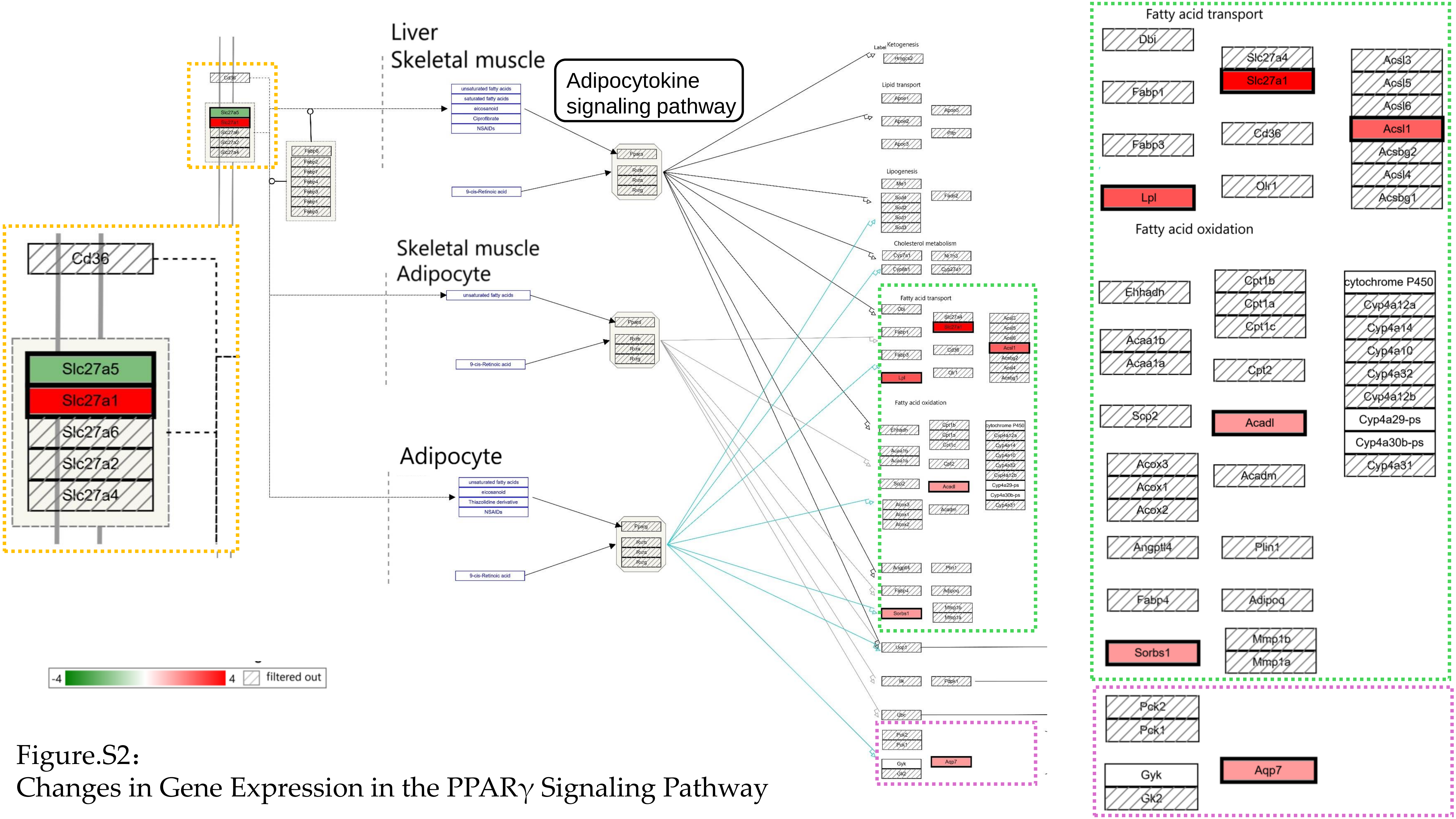

Figure.S2:
Changes in Gene Expression in the PPARγ Signaling Pathway

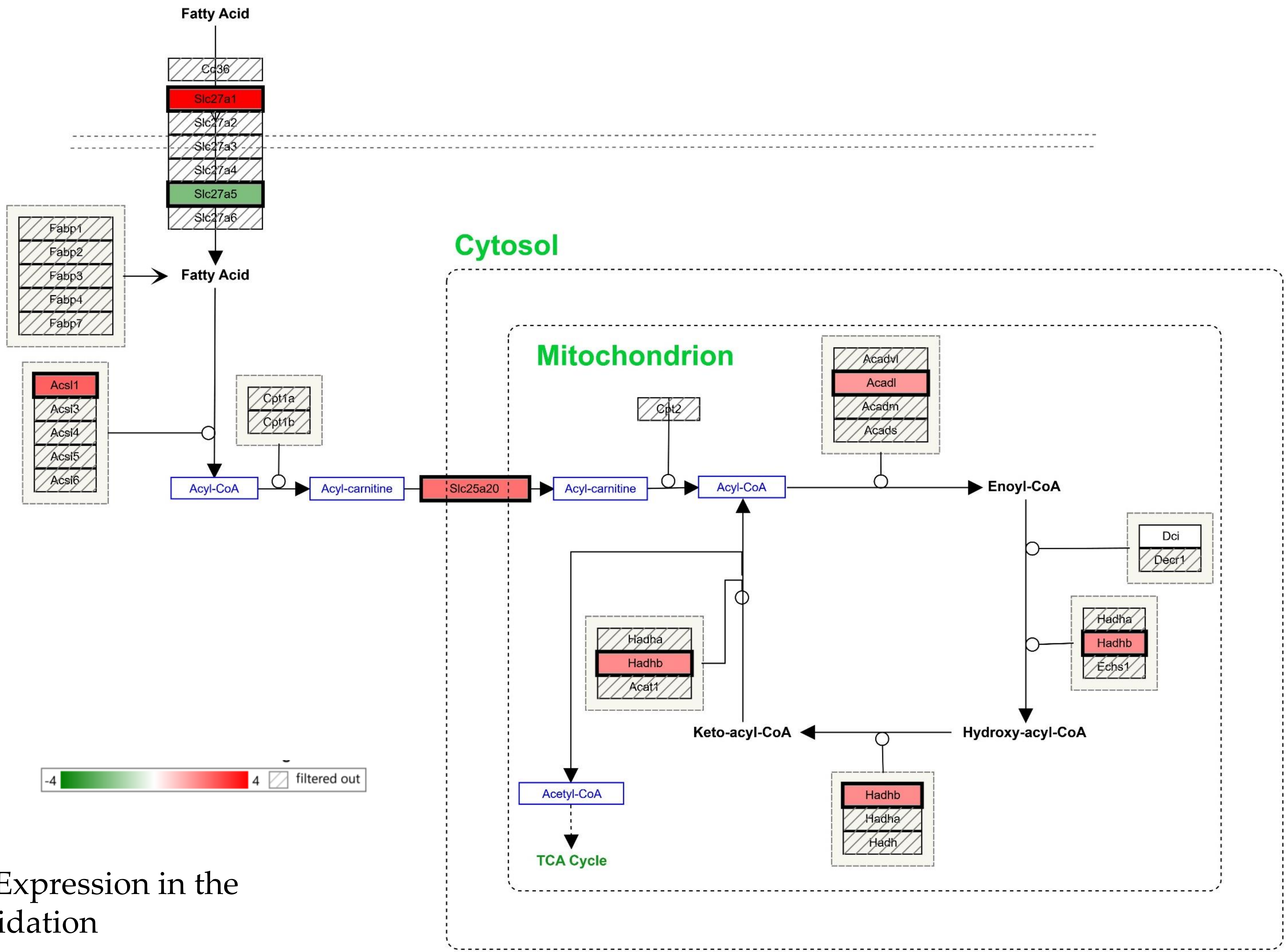

Figure.S3:
Changes in Gene Expression in the Fatty acid beta-oxidation

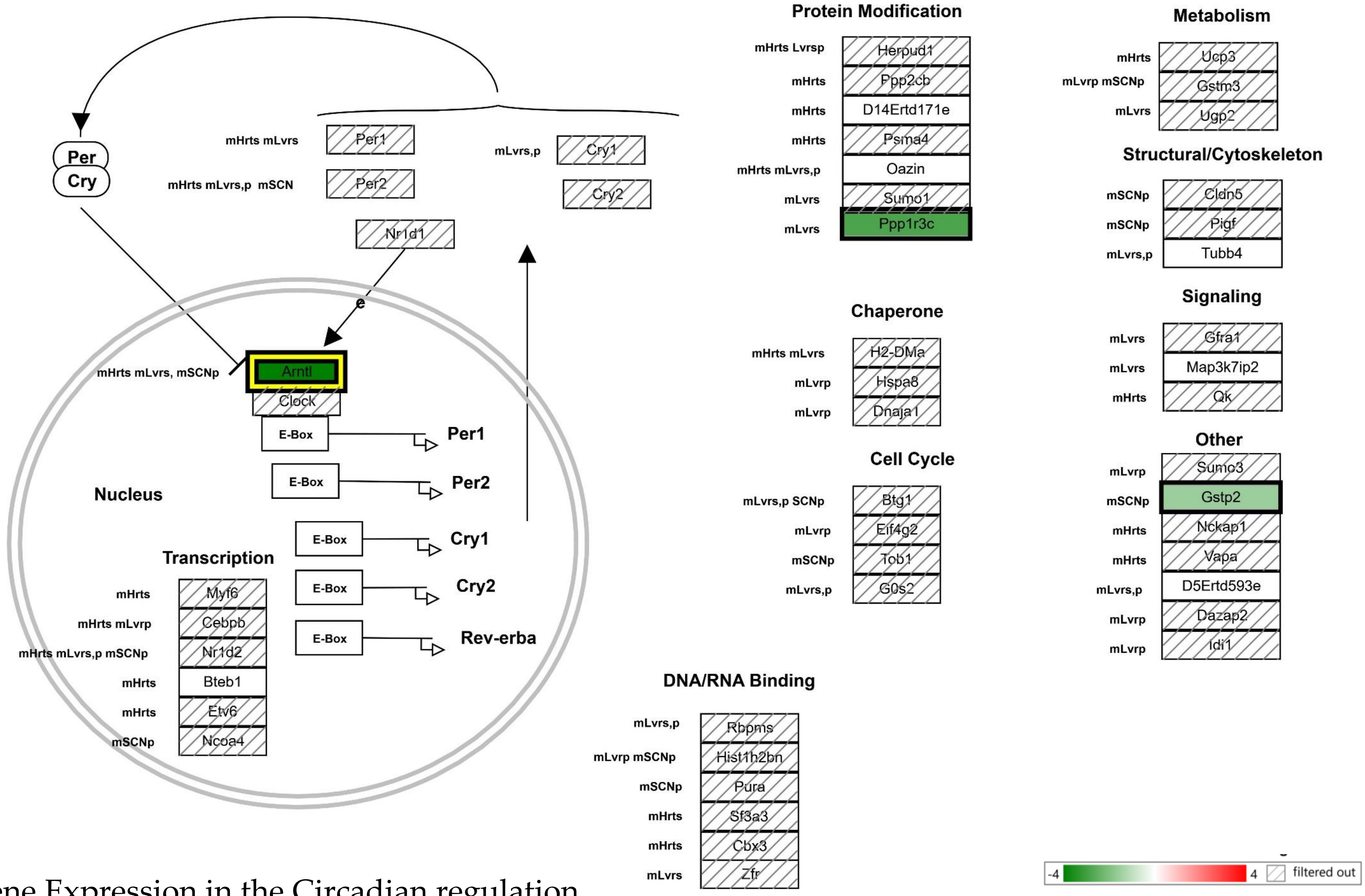

Figure.S4:
Changes in Gene Expression in the Circadian regulation
